# Nonstationary El Niño teleconnection on the post-summer upwelling off Vietnam

**DOI:** 10.1038/s41598-020-70147-2

**Published:** 2020-08-07

**Authors:** You-Lin Wang, Chau-Ron Wu

**Affiliations:** Department of Earth Sciences, National Taiwan Normal University, Taipei, Taiwan

**Keywords:** Physical oceanography, Ocean sciences, Climate-change impacts

## Abstract

Summer upwelling has often been observed off Vietnam in the South China Sea (SCS). Occasional disappearance of the upwelling has attracted much attention because it modulates the regional climate and harms surrounding fisheries. Fluctuations of the East Asian monsoon associated with El Niño are considered responsible for the weakened or abolished upwelling. However, analyses of observations performed in the present study were equivocal in terms of the dominant influence of El Niño. Based on long-term sea surface temperature data, we demonstrated that weak upwelling off Vietnam occurs more frequently during periods of accelerated global warming compared to warming hiatus periods. Warming signals in the Indian Ocean vanished relatively quickly during the hiatus period. The accompanying easterly anomalies south of the anomalous anticyclone (AAC) in the northwestern Pacific were also weakened, reducing the impact of the El Niño teleconnection on the SCS summer monsoon and thus preserving the regular post-summer upwelling off Vietnam during warming hiatus periods.

## Introduction

**Figure 1:**
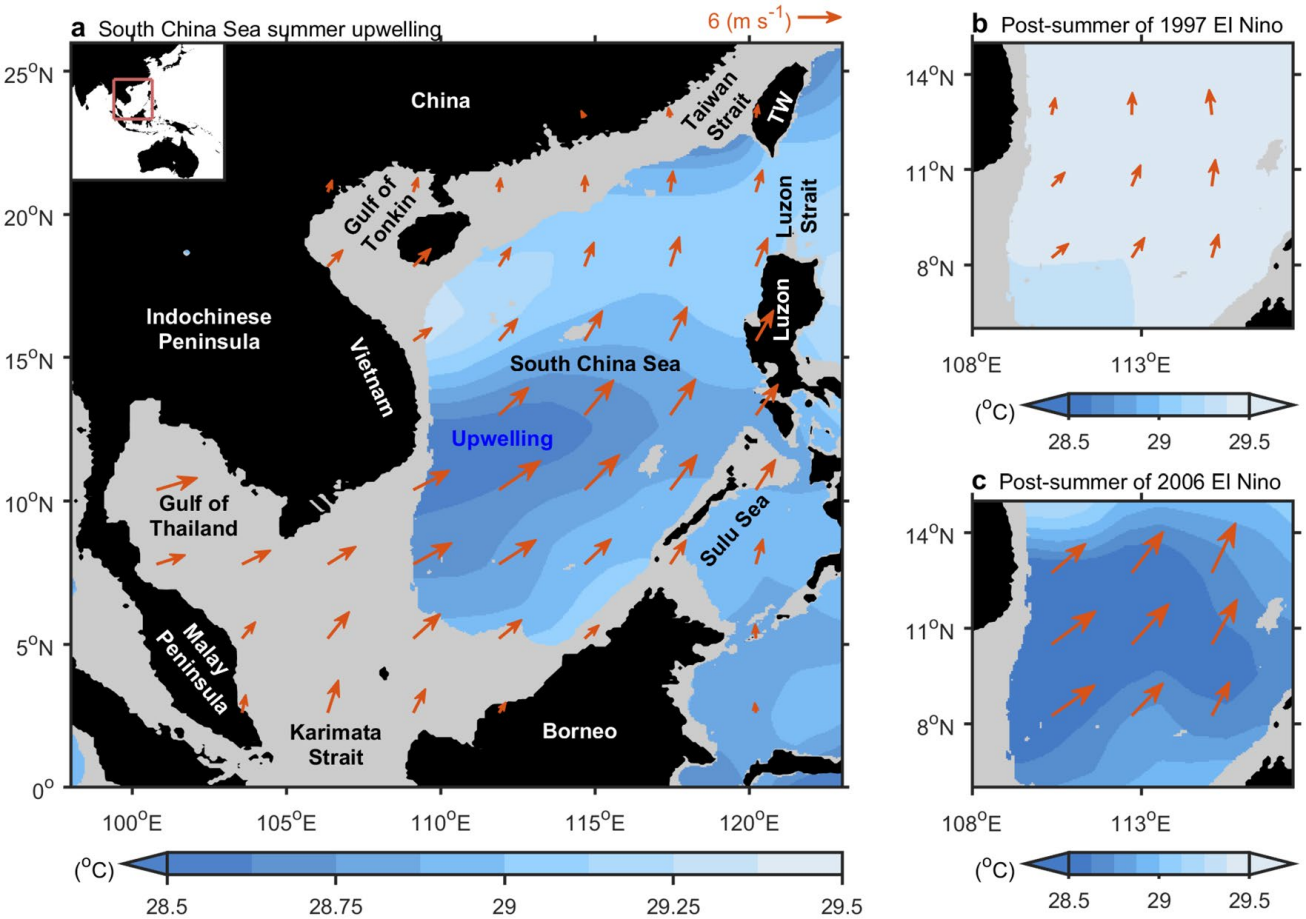
Summer upwelling in the South China Sea (SCS). (**a**) Sea surface temperature (SST, shading, 1950–2017) and surface winds (vector, 10 m wind above sea level, 1950–2017) in August. (**b**,**c**) are same as (**a**) but for the post-summer of 1997 and 2006 respectively over the upwelling region. Data are based on the HadISST and NCEPr1. Linear trend of global mean SST is removed. All figures are generated with MATLAB (R2020a, https://www.mathworks.com/).

The South China Sea (SCS), a vast marginal sea located in Southeast Asia extending from the equator to the subtropics, is heavily influenced by the seasonal reversal of the East Asian monsoon system. The SCS connects the Java Sea to the south via the Karimata Strait, the Sulu Sea to the southeast via the Mindoro and Balabac Straits, the Pacific to the northeast via the Luzon Strait, and the East China Sea to the north via the Taiwan Strait^1^ (Fig. 1). In summer, the prevailing southwesterly monsoon blows offshore off central Vietnam from May and reaches its mature phase in August, generating upwelling off Vietnam that provides nutrient-rich water and maintains local fisheries^2–5^ (Fig. 1). This wind-driven upwelling has served as an important proxy of the variability in the East Asian monsoon for more than half a century^1,2,4–12^.

During the summer of 1998, SCS upwelling off Vietnam was severely weakened, resulting in the warmest basin conditions on record, which were in turn associated with massive flooding over the Yangtze River valley^10^. The warming signal extended across the entire SCS due to the weakened summer monsoon and deeper thermocline^10^. Earlier studies attributed the weak upwelling to the delayed 1997/98 El Niño teleconnection (Fig. 1b)^2,13^. The teleconnection is associated with the anomalous anticyclone (AAC) occurring over the northwestern Pacific during the mature phase of El Niño and persists until the following summer^13^. Easterly anomalies in the southern part of the AAC diminish the southwesterly monsoon over the SCS, thereby decreasing the upwelling off Vietnam^13^. This constitutes a theoretical mechanism for the weak SCS upwelling off Vietnam caused by El Niño events. Alternatively, a recent study suggested that both the Pacific and Indian Oceans influence SCS upwelling off Vietnam, where their interaction results in a perturbation of wind direction that reduces the upwelling^6^.

However, El Niño events are not always associated with a weakened summer monsoon and SCS upwelling off Vietnam. For example, during the post-2006 El Niño summer, monsoonal winds were abnormally intense and SCS upwelling off Vietnam was obviously enhanced (Fig. 1c), indicating that the El Niño phenomenon is not solely responsible for weak SCS upwelling off Vietnam. This study aims to clarify the influence of El Niño on SCS upwelling off Vietnam during global warming and hiatus periods.

## Weak SCS upwelling events

**Figure 2:**
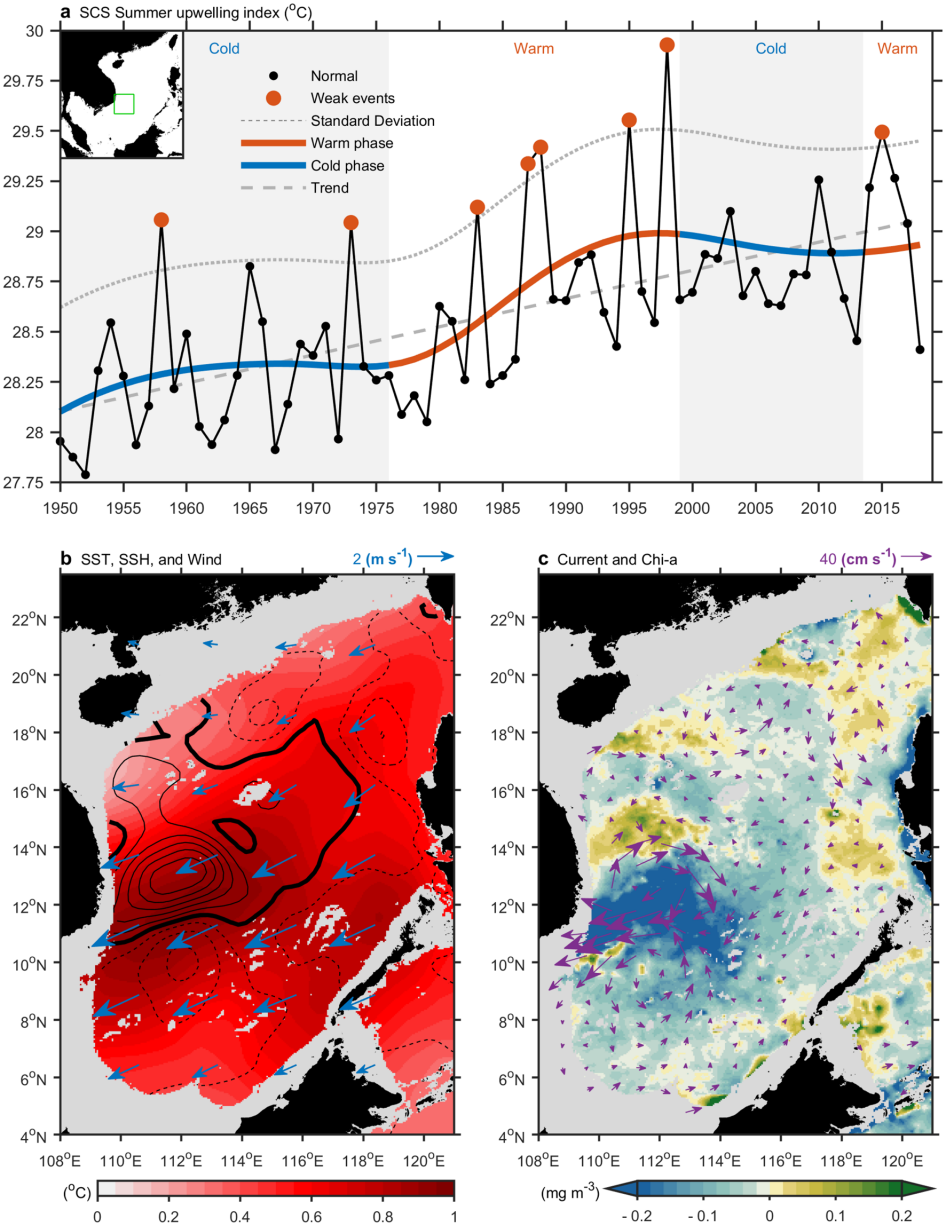
Weak SCS upwelling events. (**a**) During 1950–2018, weak events (red dots), normal events (blue dots), 1.5 standard deviation (dotted curve), warm phase (red curve), cold phase (blue curve), and trend (dashed line) of SCS upwelling are shown. (**b**) Difference (weak minus normal) of SST (shading), surface wind (vector), sea surface high (SSH, contour), and continental shelf (gray, less than 200 m of depth) are shown. Broken and thick contours indicate negative and zero SSH anomalies, respectively. Contour interval is 5 cm. (**c**) Same as (**b**) but for the difference of chlorophyll-a (shading) and current velocity (vector). Data are based on the HadISST, NCEPr1, and AVISO. All figures are generated with MATLAB (R2020a, https://www.mathworks.com/).

The sea surface temperature (SST), chlorophyll-a concentration, and sea surface height (SSH) are often used as proxies for SCS upwelling off Vietnam^2,5,10^. In this study, an ensemble empirical mode decomposition^14^ (EEMD) was performed to extract various intrinsic mode functions (IMFs). When applied to 68-year (1950–2018) SST data averaged over the upwelling region (109–113° E, 9–13° N), the EEMD yielded six IMFs. The first three IMFs are short-term fluctuations below the interannual timescale. Eight weakened summer upwelling events were identified, including 1958, 1973, 1983, 1987, 1988, 1995, 1998, and 2015 (standard deviation > 1.5; red dots in Fig. 2a). IMFs 4–6 are low-frequency components that, when summed, show the decadal variability and long-term tendency of SCS upwelling off Vietnam. In addition to the global warming signal, a phase change over a decadal timescale is apparent (bold curve in Fig. 2a). Closer inspection indicates much more frequent weak SCS upwelling events during the warm phase than during the cold phase; this phenomenon merits further study.

During weak upwelling events, the SCS summer monsoon was weakened, with a decrease in maximum wind speed of around 30–50% (− 2 to − 3 m s^−1^; mean ~ 6 m s^−1^) over the upwelling region (vectors in Fig. 2b). Under the weakened monsoon, SST anomalies increase up to 1 °C (Fig. 2b). A dipole off Vietnam with positive and negative SSHs to the north and south, respectively, indicates an anomalous westward jet (contours in Fig. 2b and vectors in Fig. 2c). This anomalous jet results in unfavorable conditions for coastal upwelling, giving rise to negative chlorophyll-a anomalies off Vietnam (shading in Fig. 2c). Warming and low-nutrient water signals are visible, extending from the coast of Vietnam to the central SCS due to weak upwelling (Fig. 2b, c).

We found that most El Niño events do not weaken summer upwelling off Vietnam in the SCS (Table 1), although almost all of the weak upwelling events took place during a post-El Niño summer. This demonstrates that SCS upwelling off Vietnam is at least partially free from El Niño control. Thus, the reportedly inescapable impact of El Niño on upwelling off Vietnam suggested by most previous studies^2,13^ should be reevaluated. The distinct evolution and dynamics of diverse El Niño events result in different climate influences on the SCS, impacting the post-summer upwelling off Vietnam.

**Table 1:** El Niño diversity and weak SCS upwelling.

Events	Period	Type	SSTA (°C)
1	1951–52	MIX	1.2
2	1953–54	MIX	0.8
**3**	**1957–58**	**CP**	**1.8**
4	1963–64	CP	1.4
5	1965–66	CP	2.0
6	1968–69	CP	1.1
7	1969–70	CP	0.9
**8**	**1972–73**	**MIX**	**2.1**
9	1976–77	MIX	0.9
10	1977–78	CP	0.8
**11**	**1982–83**	**EP**	**2.2**
**12**	**1986–87**	**MIX**	**1.2**
**13**	**1987–88**	**CP**	**1.7**
14	1991–92	CP	1.7
**15**	**1994–95**	**CP**	**1.1**
**16**	**1997–98**	**EP**	**2.4**
17	2002–03	CP	1.3
18	2004–05	CP	0.7
19	2006–07	MIX	0.9
20	2009–10	CP	1.6
**21**	**2014–16**	**MIX**	**2.6**

Period, type, and amplitude of El Niño events are shown. Bold text indicates weak SCS upwelling occurred during post-El Niño summer (according to Fig. 2a).

## Upwelling fluctuation in response to diverse El Niño events

Different El Niño events exert differential impacts on local atmospheric and oceanic circulation^15–17^. For example, regarding the impact of the 1994 and 1997 El Niño events on SCS circulation, the former enhanced the western North Pacific summer monsoon in the SCS, leading to stronger circulation in the summer of 1994. Table 1 shows that SCS upwelling may be weakened by Central Pacific (CP), Eastern Pacific (EP), or mixed-type El Niño events^18^, although relatively strong (> 2 °C El Niño events, including those in 1972/73, 1982/83, 1997/98, and 2014/16, are all associated with weak upwelling off Vietnam during the following summer. However, El Niño events do not always weaken SCS upwelling off Vietnam; in fact, most of them failed to do so during the period 1950–2018 (Table 1).

**Figure 3:**
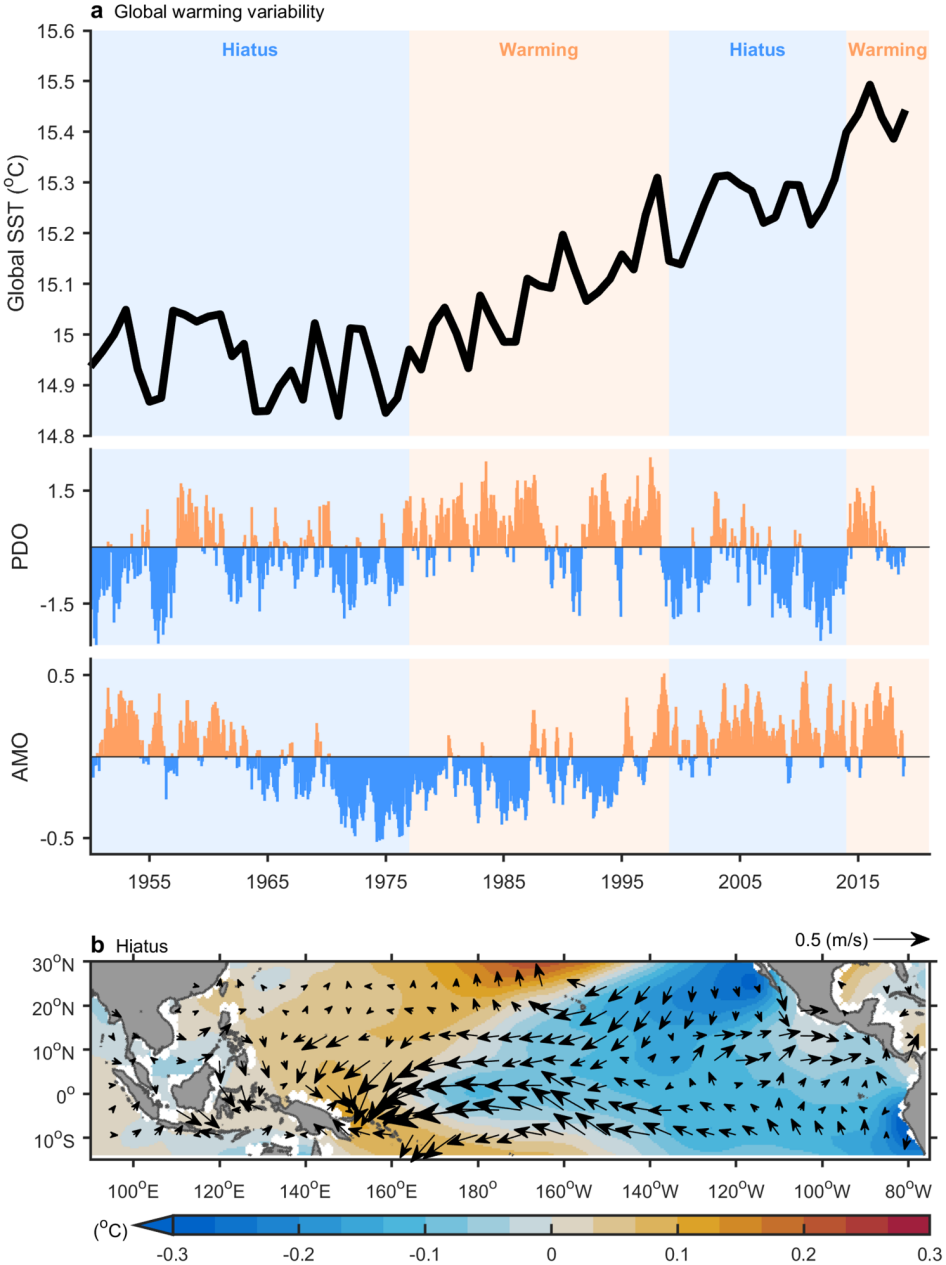
Global warming and hiatus periods. (**a**) Global averaged SST (curve), global warming acceleration periods (orange shading), global warming hiatus periods (blue shading), along with Pacific Decadal Oscillation (PDO) and Atlantic Multi-decadal Oscillation (AMO) indices. (**b**) Difference (hiatus minus warming) of SST (shading) and surface wind (vector). Data are based on the HadISST and NCEPr1. All figures are generated with MATLAB (R2020a, https://www.mathworks.com/).

**Figure 4:**
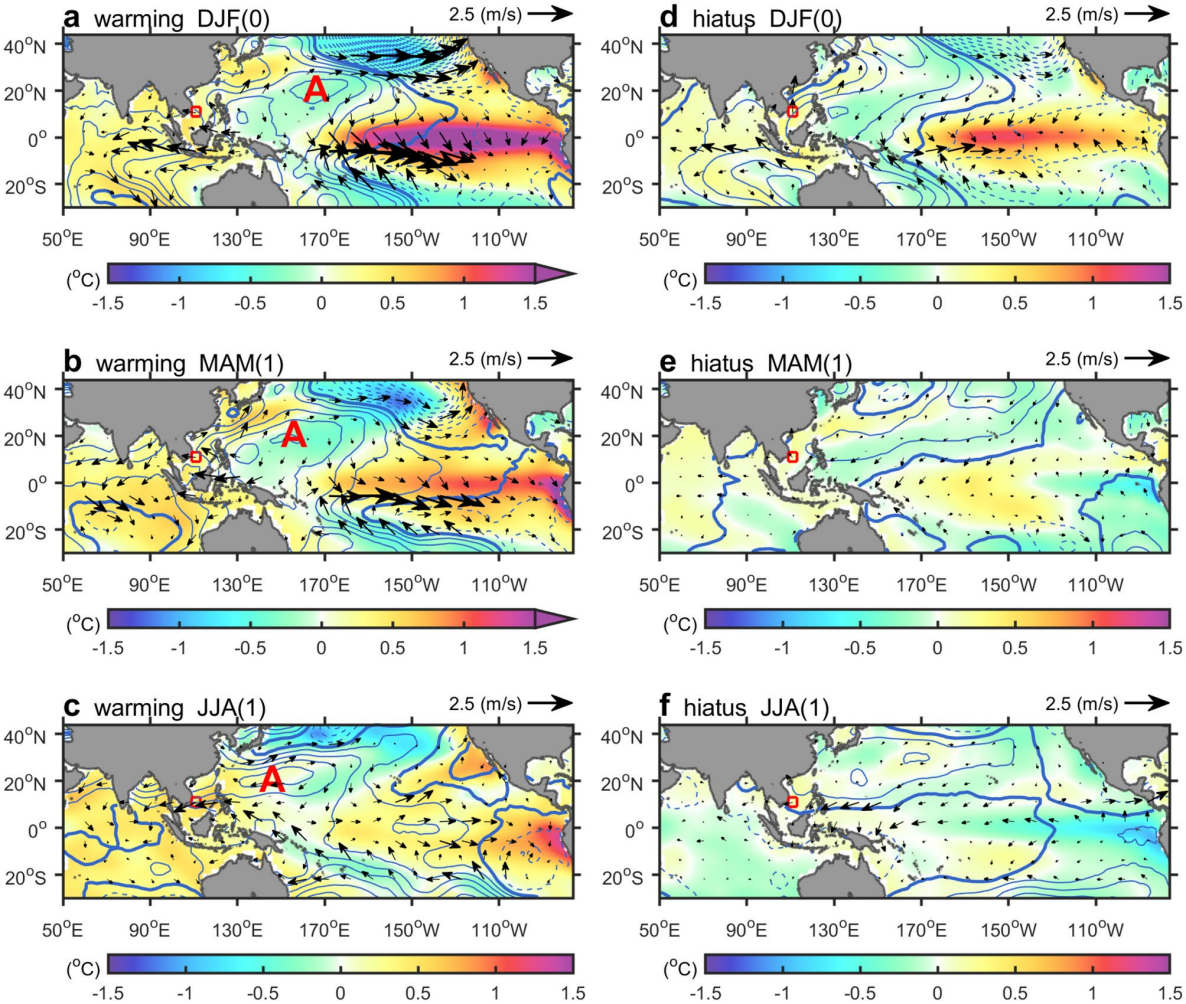
El Niño evolution and its climate influence were compared between global warming and hiatus periods. SST anomalies (shading), surface wind anomalies (vector), and sea level pressure anomalies (contour) are displayed in (**a**) El Niño mature phase (December–February), (**b**) post-spring (March–May), (**c**) post-summer (June–August) during global warming acceleration period. (**d**–**f**) Same as (**a**–**c**) but for warming hiatus period. SCS upwelling region (red box) and the anomalous anticyclone (marked by an “A”) are also indicated. Dash, thin, and thick contours express negative, positive, and zero anomalies, while contour interval is 0.25 hPa. All figures are generated with MATLAB (R2020a, https://www.mathworks.com/).

Furthermore, SCS upwelling off Vietnam shows decadal variability. Weak SCS upwelling off Vietnam often occurred during El Niño events in the period 1978–1998 and after 2014 (Fig. 2a^,^Table 1). These two periods correspond to periods of accelerated global warming (Fig. 3a) and were characterized by generally stronger El Niños (Table 1). By contrast, few weak upwelling events were identified during El Niño events prior to 1977, or in the period 1999–2013; both of these periods were characterized by a warming hiatus (Fig. 3a). Global warming hiatuses are attributed to natural variability to some extent^19–22^, including fluctuations of Pacific Decadal Oscillation (PDO)^21^ and/or Atlantic multidecadal oscillation (AMO)^20^ (Fig. 3a). During the regime shift of the PDO/AMO since the late 1990s, warming over the North Atlantic triggers transbasin teleconnections that lead to stronger trade winds and cooling over the eastern and central Pacific (Fig. 3b)^20,23^. Those conditions contribute to the global warming hiatus and cause a La Niña-like decadal cooling^22^ which do not favor the development of El Niño events. The strong association between El Niño and SCS upwelling off Vietnam has deteriorated sharply during the hiatus period (Table 1) because of weakened El Niño events. The dissimilar El Niño dynamics between global warming and hiatus periods leads to different atmospheric and oceanic anomalies in the northwestern Pacific, which exert an immediate impact on SCS upwelling off Vietnam. To further examine the influence of climate, the evolution of El Niño was compared between warming and hiatus periods (Fig. 4).

## Mechanism

SST and surface wind anomalies indicate that the mature-phase El Niño during the hiatus period is substantially weaker in the eastern and central tropical Pacific compared to that during the warming period. This is illustrated in Fig. 4a,d: SST anomalies over the tropical Pacific are reduced from 1.5–2 °C to 0.5–1 °C, and maximum westerly anomalies decrease from ~ 2.5 to ~ 1 m s^−1^. Notably, high-pressure anomalies, such as the AAC (marked by an “A” in Fig. 4a), which are usually seen in the northwestern Pacific during the mature phase of El Niño^13^ vanish during the hiatus phase (Fig. 4d).

SST and westerly anomalies in the tropical Pacific are diminished during the post-El Niño spring in both warming and hiatus periods (Fig. 4b,e). In particular, the signatures of El Niño events, including SST, surface wind, and sea level pressure anomalies, are largely absent in the Pacific and Indian Oceans during the post-El Niño spring in the hiatus period (Fig. 4e), indicating that the lifetime of El Niño events during the hiatus period is much shorter than that during the warming period.

In the post-El Niño summer, a noteworthy feature distinguishing warming and hiatus periods is the AAC over the northwestern Pacific (Fig. 4c,f). The AAC is apparent only during the warming period El Niño event. This mature AAC is associated with easterly anomalies on its southern border that decrease the southwesterly monsoon in the SCS, thus weakening upwelling in the summer. The easterly anomalies to the south of the AAC are maintained by the El Niño teleconnection that occurs due to warming of the Indian Ocean^13^, which stores the warming signals from the previous El Niño. Regarding the underlying dynamics, El Niño triggers oceanic Rossby waves in the eastern Indian Ocean, which propagate warming signals westward in winter, leading to warming in the Indian Ocean during the post-El Niño spring and, in turn, easterly anomalies south of the AAC in the summer^13^. By contrast, the warming signals in the Indian Ocean vanish quickly during the El Niño in the hiatus period (Fig. 4d–f). The accompanying easterly anomalies south of the AAC are further weakened, removing any influence of El Niño on the SCS summer monsoon and the associated upwelling off Vietnam during the post-El Niño summer.

In summary, the comparatively weak and short El Niño events during the hiatus period do not induce warming over the Indian Ocean during the post-El Niño summer. The reduced warming of the Indian Ocean weakens the AAC over the northwestern Pacific, thus decreasing the impact of El Niño on the SCS summer monsoon and the associated upwelling.

## Concluding remarks

In this study, we compared the effects of El Niño events on SCS upwelling off Vietnam between warming and hiatus periods. We identified weak SCS upwelling events based on 68 years of observations. Most El Niño events do not weaken summer SCS upwelling off Vietnam, although almost all of the weak upwelling events occurred during a post-El Niño summer. The distinct evolution and dynamics of different El Niño events lead to differential impacts on the SCS, including on summer upwelling. Furthermore, SCS upwelling off Vietnam shows decadal variability. Weak SCS upwelling often occurs during El Niños in periods of global warming, and is usually accompanied by stronger El Niños. By contrast, few weak upwelling events were identified during El Niño events in warming hiatus periods. El Niño events during the hiatus period are substantially weaker in the eastern and central tropical Pacific compared to those during warming periods. Warming signals in the Indian Ocean vanish quickly during El Niño events in warming hiatus periods. The accompanying easterly anomalies south of the AAC are also weakened, thus removing any influence of the El Niño event on the SCS summer monsoon, and on the associated upwelling during the post-El Niño summer in warming hiatus periods.

## Methods

Monthly 1° × 1° SST data of the Hadley Centre Sea Ice and Sea Surface Temperature product (HadISST)^24^ are provided by the Hadley Centre/Met Office (https://www.metoffice.gov.uk/hadobs/). Grids of SSTs where data are incomplete in temporal are ignored. Daily 0.25° × 0.25° SSH and geostrophic velocity (a version of Ssalto/Duacs DT-MADT two-sat)^25^ are adopted from the Archiving, Validation, and Interpretation of Satellite Oceanographic data (AVISO) (https://www.aviso.altimetry.fr). Daily 1.875° × 1.875° 10 m wind velocity and 2.5° × 2.5° sea level pressure of the National Centers for Environmental Prediction reanalysis 1 (NCEPr1)^26^ are provided by the National Oceanic and Atmospheric Administration/National Centers for Environmental Prediction (NOAA/NCEP) (https://www.ncep.noaa.gov/). Daily chlorophyll-a concentration data of the SeaWiFS and the MODIS are given by National Aeronautics and Space Administration (NASA)/Ocean Color (https://oceancolor.gsfc.nasa.gov/).

The definition of El Niño diversity in Table 1 is based on Yu and Kim^18^ and Paek et al.^27^. The PDO and AMO indices are adopted from the Joint Institute for the Study of the Atmosphere and Ocean (JISAO) and the NOAA/Earth System Research Laboratory (ESRL), respectively.
